# *Halyomorpha halys* (Hemiptera: Pentatomidae) as the major contributor to early olive drop in northern Italy

**DOI:** 10.1093/jee/toae126

**Published:** 2024-06-13

**Authors:** Francesco Sanna, Nicola Mori, Giacomo Santoiemma, Alberto Pozzebon, Davide Scaccini, Federico Marangoni, Luca Sella

**Affiliations:** Department of Agronomy, Food, Natural Resources, Animals and Environment (DAFNAE), University of Padova, Legnaro, Padova, Italy; Department of Land, Environment, Agriculture and Forestry (TESAF), University of Padova, Legnaro, Padova, Italy; Department of Biotechnology, University of Verona, Verona, Italy; Department of Agronomy, Food, Natural Resources, Animals and Environment (DAFNAE), University of Padova, Legnaro, Padova, Italy; Department of Agronomy, Food, Natural Resources, Animals and Environment (DAFNAE), University of Padova, Legnaro, Padova, Italy; Department of Agronomy, Food, Natural Resources, Animals and Environment (DAFNAE), University of Padova, Legnaro, Padova, Italy; Department of Biotechnology, University of Verona, Verona, Italy; Department of Land, Environment, Agriculture and Forestry (TESAF), University of Padova, Legnaro, Padova, Italy

**Keywords:** brown marmorated stink bug, fungal olive disease, Heteroptera feeding damage, integrated stink bug management

## Abstract

In recent years, a new phenomenon of early olive drop is causing production losses in olive groves throughout northern Italy. To analyze the possible causes, field and laboratory trials were performed to assess the involvement of fungal pathogens and insect pests in this disease. External and internal symptoms of fungal infections or insect-feeding activities were researched. Fungi present in healthy and dislodged olives were investigated. The relationship between olives that fell and *Halyomorpha halys* (Stål) (Hemiptera: Pentatomidae) infestation was assessed in a controlled infestation trial, and the effectiveness of an insecticidal strategy in reducing early olive drop was tested in open field conditions. A comparable number of fungi, mostly endophytes, were isolated and identified from both healthy and dislodged olives. The damage observed on dislodged olives was primarily ascribed to pentatomids feeding activity. Six stink bugs species were found in olive canopies, that is, the invasive *H. halys*, which was by far the most abundant, and *Acrosternum heegeri* Fieber, *Nezara viridula* (Linnaeus), *Palomena prasina* (Linnaeus), *Piezodorus lituratus* (Fabricious), and *Rhaphigaster nebulosa* (Poda)*. Halyomorpha halys* caused intense fruit drop in the controlled infestation trial, and its infestation level significantly correlated with the number of olives that fell. Native stink bugs, present in much lower population compared to *H. halys*, could also partially contribute to early drop of olives. Insect proof net significantly reduced the early olive drop disease, while insecticide applications only partially reduced the stink bugs population density and, proportionally, early olive drop.

## Introduction

Olive (*Olea europaea* Linnaeus, 1753) is a fruit tree native to Minor Asia and Syria and it is cultivated in Mediterranean countries, where over 95% of worldwide olives are produced (Bartolini and Petrucelli 2002, FAOStat 2022). Italy is the second producer worldwide, with over 1 million ha of olive groves (ISTAT 2021, European Commission 2024).

Olive trees are subject to different abiotic and biotic factors that can affect the quantity and the quality of olive production, in particular from flowering to fruit harvest. Among abiotic issues, physiological disorders and direct damage to both olive trees and fruits can be caused by multiple atmospheric and pedoclimatic conditions like high temperatures, drought, hailstorms, wind, pollutants, waterlogging, nutrient deficiency, as well as by other causes (Sanzani et al. 2012). On the other hand, also biotic factors may increase fruit losses or cause deterioration in quality and are mainly caused by insects and fungi. Considering insects, the 2 main olive pests are the olive fruit fly *Bactrocera oleae* (Rossi) (Diptera: Tephrytidae) (Daane and Johnson 2010) and the olive moth *Prays oleae* Bernard (Lepidoptera: Praydidae) (Ramos et al. 1998). *Bactrocera oleae* lays its eggs inside the fruits from stone hardening, and larvae develop by feeding on the olive pulp. High *B. oleae* infestations may occur in autumn, during fruit maturity stages, when maximum daily temperature no longer exceeds 30–32 °C, preventing the development of eggs and larvae, and the higher humidity enhances adult survival (Malheiro et al. 2015, Marchini et al. 2017). Similarly, *P. oleae* attacks olives at the beginning of summer, during fruit development, with larvae penetrating the fruit to feed on its pulp. Once mature—between Sep and Oct—larvae emerge from the fruit causing olive drop (Ramos et al. 1998).

Furthermore, intense fruit losses and decays are caused by fungal diseases such as the olive anthracnose (soft rot and mummification of olives caused by *Colletotrichum* species) (Cacciola et al. 2012, Talhinhas et al. 2018, Peres et al. 2021) and the Dalmatian disease (sunken, necrotic, and circular lesions caused by *Botryosphaeria dothidea*), which are strongly related to wounds made by the olive fruit fly in autumn and winter (Iannotta et al. 2007, Lazzizera et al. 2008, Moral et al. 2010, 2019). Other minor fungal pathogens in olive groves are *Alternaria alternata*, *Aspergillus* spp., *Fusarium* spp., *Penicillium* spp., and *Pseudocercospora cladosporioides* that, similarly to *Colletotrichum* spp. and *B. dothidea*, can cause olive decays especially late in the season—during fruit ripening—when relative humidity is high and damage by *B. oleae* is frequent (Angerosa et al. 1999, Moral et al. 2008, Markakis et al. 2021).

In recent years, increased levels of olive drop have been reported in northern Italy (Zapponi et al. 2022, Linaldeddu et al. 2023). From field observations, fruit drop can be observed from Jun to early Aug, during fruit development (Linaldeddu et al. 2023). The occurrence of nonspecific symptoms has led to the formulation of various hypotheses to explain this disease, including fungal pathogens (Linaldeddu et al. 2020, 2023) and the feeding activity of a recently introduced pest, the brown marmorated stink bug *Halyomorpha halys* (Stål) (Hemiptera: Pentatomidae) (Zapponi et al. 2022, Daher et al. 2023). The latter has been shown to cause olive drop when confined to fruiting branches at very high density, especially before stone hardening (Zapponi et al. 2022, Daher et al. 2023).

In this study, the role of fungi and insects in causing early olive drop was investigated. Healthy and dislodged olives sampled from numerous olive groves were examined to detect external and internal symptoms of fungal infections or insect-feeding activities. Fungi were isolated and identified, and the insect community in olive tree canopies was investigated. Furthermore, damage assessment caused by *H. halys* feeding activity at different infestation density under confined conditions was performed. Finally, the effectiveness of an insecticidal strategy in reducing early olive drop was investigated in open field conditions.

## Materials and Methods

### Study Area

Surveys and trials were conducted from 2021 to 2023 in up to 11 olive groves located in Veneto region, northern Italy (Table 1). The area is located at the northern edge of olive tree distribution, and groves are patchily occurring in hilly areas or lakesides, intermixed with woods and vineyards. The olive groves ranged from 21 to 227 m above sea level, had heterogeneous sizes (from 0.2 to 2 ha), and were characterized by the presence of local, national, and international cultivars (Table 1) whose ages ranged from 8 to more than 50 years. In all the olive groves, the ground cover was characterized by permanent grass that was periodically mowed during the 3 growing seasons. All were managed according to the integrated pest management guidelines of the Veneto Region (2021, 2022, 2023). For each site, pesticide applications and other details are reported in Table 1.

**Table 1.** Investigated olive groves

Code	Coordinates	Area (ha)	Olive varieties	Activities	Phytosanitary applications carried out by farmers before or during activities.
G1	45.266804, 11.703367	1.4	Leccino**, Pendolino**, Rasara*	Fs, Ci	/
G2	45.258973, 11.707715	0.4	Grignano*, Leccino**, Pendolino**	In	Trifloxystrobin 62.5 g ha^−1^ and tebuconazole 125 g ha^−1^ (BBCH57)
G3	45.275054, 11.727569	1.1	Grignano, Leccino, Pendolino, Rasara	In	Trifloxystrobin 62.5 g ha^−1^ and tebuconazole 125 g ha^−1^ (BBCH57)
G4	45.270644, 11.740407	1.8	Grignano, Leccino, Pendolino, Rasara	In	Trifloxystrobin 62.5 g ha^−1^ and tebuconazole 125 g ha^−1^ (BBCH57)
G5	45.406630, 11.538871	0.3	Frantoio**, Leccino, Leccione**, Pendolino	Fs, Ci, In	Spinosad bait 0.24 g ha^−1^ (BBCH75 onwards)
G6	45.534175, 10.736419	0.6	Casaliva*, Grignano	Fs	/
G7	45.441256, 11.590452	1.5	Arbecchina***, Arbosana***, Favolosa**, Leccino	Fs, In	/
G8	45.542072, 10.751595	1.1	Casaliva, Grignano	Fs, Ci	/
G9	45.456556, 11.103417	2.0	Casaliva, Favarol*, Grignano	Fs, Ci	Acetamiprid 100 g ha^−1^ (BBCH72)
G10	45.423874, 11.432144	1.6	Casaliva, Frantoio, Grignano, Leccino, Trepp*	Fs, Ci	Spinosad bait 0.24 g ha^−1^ (BBCH75 onwards)
G11	45.315641, 11.772327	0.2	Favarol, Grignano, Leccino, Rasara	Fs	Trifloxystrobin 62.5 g ha^−1^ and tebuconazole 125 g ha^−1^ (BBCH57), difenoconazole 125 g ha^−1^ (BBCH71)
G12	45.416475, 11.536983	0.5	Grignano, Favarol, Frantoio, Leccino, Pendolino, Trepp	Fs, Ci	Spinosad bait 0.24 g ha^−1^ (BBCH75 onwards)
G13	45.465222, 11.119056	0.5	Casaliva, Favarol, Grignano	Fs, Ci	Acetamiprid 100 g ha^−1^ (BBCH72)
G14	45.400595, 11.506597	0.5	Frantoio, Grignano, Leccio del corno**, Leccino, Pendolino	Fs	/

Fs: field survey, Ci: controlled infestation, In: insecticide experiment. Olive varieties column: * = local, ** = national, and *** = international variety.

As plant development over time can differ annually and across locations, the olive phenological stage was used in this research to determine the timing for each activity. Plant phenology was presented according to the phenological scale officially accepted by the European and Mediterranean Plant Protection Organization (EPPO), the BBCH (Biologische Bundesantstalt, Bundessortenamt, Chemische Industrie) scale (Sanz-Cortés et al. 2002).

### Field Surveys of the Phytosanitary Status of Olives and of Heteroptera Community in Olive Canopies During the Early Drop Period

In 2021, a preliminary characterization of the phytosanitary status (as symptoms of fungal infections or insect feeding activities) was performed in 3 olive groves (G1, G6, and G8; Table 1). In total 540 (200 olives each in G6 and G8, 140 in G1) dislodged olives (i.e., fruits detaching from plants when touched or by gently shaking branches) were collected in mid-Jul, when they were at BBCH 73–74 (fruits between 30% and 40% of final size). Olives were stored at 4 °C and analyzed within 24 h for both internal and external necrosis or insect-borne damage under a stereomicroscope (Wild, Heerbrugg, Switzerland, model M3C, 6.4-40x), with the help of a surgical scalpel.

Based on the results obtained in 2021, the second-year surveys took place between 8th and 17th Jul 2022 (BBCH72-74, fruits between 20% and 40% of final size) in 11 olive groves (Table 1). The Heteroptera feeding activities on dislodged vs. healthy olives, the fungi colonizing olives, and the Heteroptera community in olive tree canopies, were investigated.

**Fig. 1.**
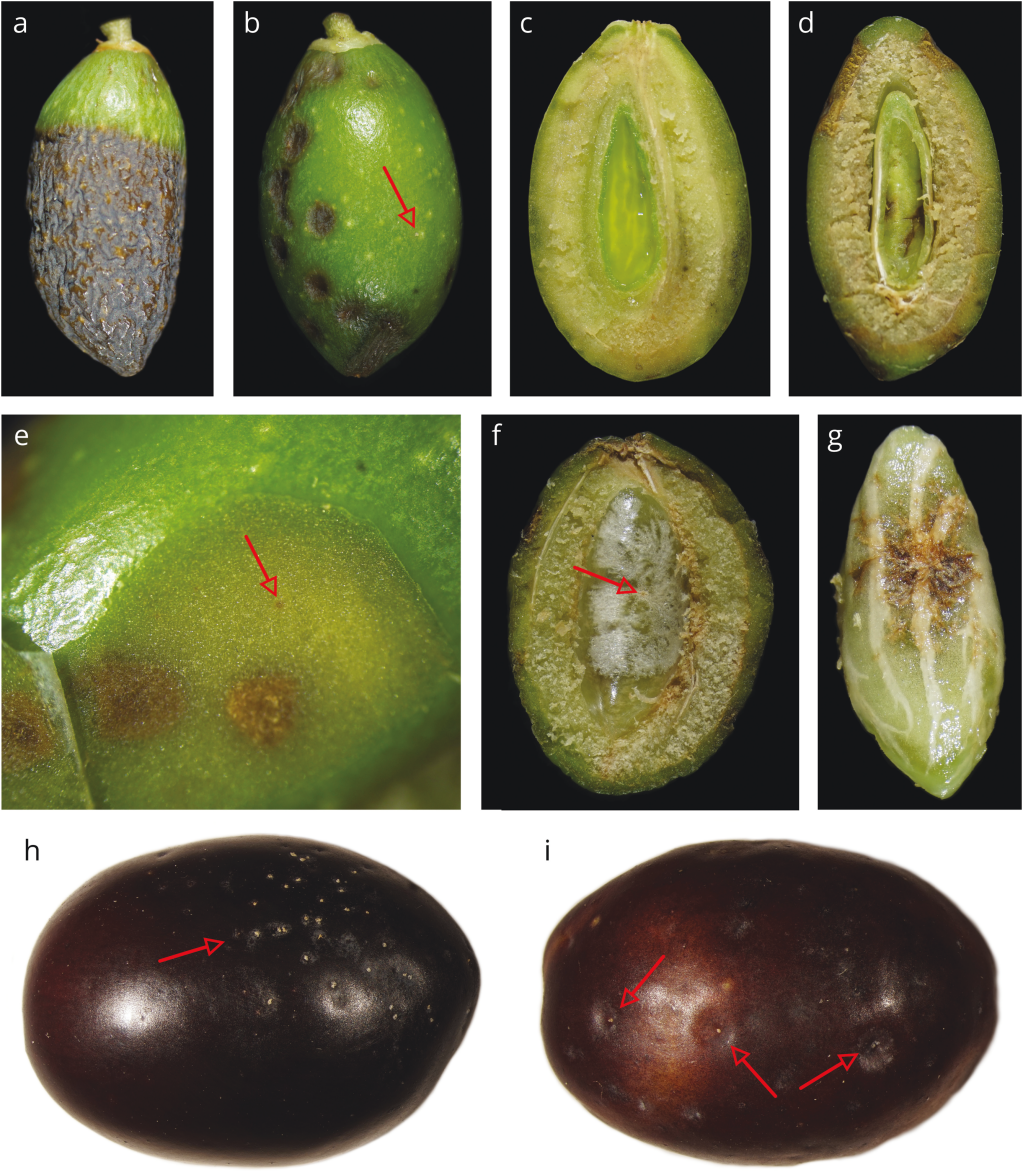
(A, B) External symptoms of the early olive drop disease; (C) olive with healthy seed; (D) olive with necrotic seed emptied of its endosperm; (E) *Halyomorpha halys* feeding mark on the olive pulp; (F) effect of *H. halys* feeding activity on the internal membrane of endocarp; (G) seed necrosis caused by *H. halys*; (H, I) salivary sheaths on olives. Arrows indicate *H. halys* feeding wounds.

Heteroptera feeding activities were characterized by 10 dislodged and 10 healthy olives randomly picked from 10 plants (200 fruits per grove), regardless of the cultivar. Dislodged olives were sampled as described for the preliminary activity performed in 2021, while the healthy olives, consisting in fruits that were firmly attached to the branches, were manually picked. Olives were stored and inspected for Heteroptera feeding wounds as done in 2021. The presence of feeding activity was confirmed when the endosperm of the fruit was lacking, and by the presence of seed necrosis, pericarp whitening, and feeding wounds, often associated with visible salivary sheaths (Fig. 1B–G) (Panizzi 1997, Peiffer and Felton 2014).

Fungal community was investigated in 10 dislodged vs. 10 healthy olives randomly collected from each of the 11 groves (220 total olives). Olives were sterilized on their surface by dipping them in 70% ethanol for 10 s, washed thoroughly in sterile water, dried under a laminar flow hood, deprived of style residues, and cut in half with a sterile scalpel. Petioles, pericarps, and seeds were plated separately on 90 mm Petri dishes containing 10 ml of Potato Dextrose Agar (PDA, 39 g l^−1^, Difco Laboratories, Detroit, MI, USA), and incubated at 25°C for 2 wk. Grown fungal colonies were isolated and identified based on morphological features following Goidanich (1964) and Doveri (2013). In case the morphological identification was not straightforward, molecular identification of fungal isolates was carried out by extracting fungal DNA from fresh mycelia with the Dneasy Plant mini kit (Qiagen, Hilden, Germany). DNA was then used as a template in a PCR reaction with the universal primers ITS1 and ITS4 (White et al. 1990) to amplify the internal transcribed spacer regions of ribosomal DNA (ITS-rDNA). Amplicons were then purified and subjected to sequencing. The sequence of each isolate was compared to the available sequences in the GenBank database by using BLAST (http://blast.ncbi.nlm.nih.gov).

Heteroptera in canopies were investigated through direct collection using a sweeping entomological net (Entosphinx, Pardubice, Czech Republic; ring diameter: 35 cm) between 1 and 3.5 m canopy height. Five sweepings were performed on the plant where dislodged and healthy olives were sampled and twenty on the surrounding plants, sampling 10 different points. A total of 250 sweeps per olive grove were carried out.

### Damage Assessment of *H. halys* on Olives

To investigate the role of *H. halys* in olive early drop, trials took place in 7 groves (Table 1) during early fruit development (i.e., before hardening of the stone) and from late fruit development up to harvest maturity in 2022. In each grove, 4 treatments were tested: (i) branch with a single adult of *H. halys* confined (male or female, maintaining the sex ratio as close as possible to 50%); (ii) branch with 2 adults of *H. halys* confined (1 male and 1 female); (iii) branch with 4 adults of *H. halys* confined (2 males and 2 females); (iv) branch with no *H. halys* individuals confined.

For both early and late fruit development trials, insect infestation was managed by installing insect-proof net sleeves of 70 × 100 cm, mesh 0.8 mm, on fruiting branches of about 0.1 m^3^. The 4 treatments were arranged in 1 tree in each olive grove. In total, we set up 56 (2 timing × 4 treatment × 7 sites) cages. Sleeves included at least 100 fruits and were placed at the end of Jun (BBCH71-72, fruits between 10% and 20% of final size). After that, the early fruit development experiment started on 14th Jul (BBCH73-74), while the late fruit development one on 2nd Sep (BBCH78-79, fruits between 80% and 90% of final size). *Halyomorpha halys* individuals were left inside the sleeves for 3 days in the case of early fruit development and for a month in the late fruit development experiment. The long exposure time of olives to *H. halys* in the second experiment was made to simulate the potential continuous infestation, as often occurs in the field. Sleeves were inspected every 3 days for a 15-day period in the early fruit development experiment, and weekly until harvesting in the late fruit development one. In each sampling event, fallen olives were counted and analyzed searching for seed necrosis, stink bug feeding wounds, and other symptoms ascribed to *H. halys* presence. In the early fruit development experiment, sleeves presenting dead stink bugs (3 in total, 1 in the treatment with a single *H. halys* adult and 2 in the treatment with 2 *H. halys* individuals) were excluded from the trial. In the late fruit development experiment, the dead insects were just replaced at each checking of the sleeves.

### Effectiveness of Insecticidal Strategy in Reducing Early Olive Drop and Stink Bug Population in Open Field Conditions

Field trials were performed in 2023 in 5 olive groves (Table 1). In each olive grove, 2 insecticide applications were performed during phenological phases sensitive to early olive drop—the first in mid-Jun (BBCH69, fruit set) and the second at the end of Jun to the beginning of Jul (BBCH72), with a 15-day interval between them.

As insecticides, deltamethrin and acetamiprid were applied, considering their registration on olive groves (Veneto Region 2023) and their efficacy against *H. halys* (Leskey et al. 2012, Bergmann and Raupp 2014, Kuhar and Kamminga 2017, Preti et al. 2018). Selected commercial formulations were Decis® EVO (Bayer, Leverkusen, Germany), applied at 0.7 L ha^−1^ (17.5 g ha^−1^ of active substance) for the first application, and Epik® SL (SIPCAM OXON, Lodi, Italy), applied at 1.5 L ha^−1^ (75 g ha^−1^ of active substance) for the second one. Application volume was equivalent to 800 L ha^−1^. As negative control, at least 25 trees of each olive grove were left unsprayed. Physical exclusion of stink bugs by using insect-proof net sleeves of 70 × 100 cm (mesh 0.8 mm) was also included as positive control within the sprayed and unsprayed plots, to assess the early olive drop in complete absence of stink bugs. During the insect-proof caging period, the protected branches were carefully inspected to remove the new-born or unseen pentatomids possibly present. It should be underlined that the use of cages was considered as positive control because none of the insecticides can ensure a complete and lasting elimination of stink bugs (Kuhar and Kamminga 2017, Preti et al. 2018).

Olive drop was monitored by periodically counting the olives remaining in the selected fruiting branches on 4 plants per plot (8 per grove). On each plant, drupes were counted and monitored in 2 adjacent branches of about 0.15 m^3^, one protected and one unprotected by insect-proof net. To skip most of ovary and fruit abscission directly related to fruit set (Rallo and Fernández-Escobar 1985) and to cover the entire period of early drop of olives (Linaldeddu et al. 2023), sampling was performed from BBCH71 (about 20 days after full bloom) to BBCH79 every 11 ± 4.5 days (mean ± SD), for a total of 7 samplings.

Moreover, infestation of *H. halys* and other stink bug species was assessed before the 1^st^ insecticide application and then from BBCH71 to BBCH79, concurrently with olive drop quantification. For each plot, ten replicates of 10 sweeps (200 total sweeps per grove) were carried out on olive canopies with an entomological sweeping net, with the methodology described before.

### Data Analysis

Data analysis was performed with R software (R Core Team 2020). Effects of health status (dislodged vs. healthy), fruit part (seed, pericarp, and petiole), and their interaction were explored on fungal colonies (presence/absence) that emerged from olives with generalized linear mixed models (GLMMs) with a binomial distribution (logit link-function). All the explanatory variables were included in the models as categorical variables. Olive grove identity was included as a random factor. The analysis was performed using the ‘lme4’ package (Bates et al. 2017). Overdispersion and residual distribution were checked using the ‘DHARMa’ package (Hartig 2017). Pairwise comparisons were performed using adjusted *P*-values (Tukey method) using the ‘emmeans’ package (Lenth et al. 2020).

Effects of feeding of *H. halys* in the damage assessment trials, as well as in the open field trial with treatments (sprayed vs. unsprayed vs. protected by net), were explored on olives dropped over time with marginal Cox survival models using the ‘survival’ package (Therneau 2022). Robust standard errors were applied to account for possible intra-cluster dependence due to the different tree branches (Therneau and Grambsch 2000, Martinussen and Scheike 2007). The dependent variable was the time until the drop of each olive. All the explanatory variables (number of stink bugs, phytosanitary applications, physical exclusion) were included as categorical variables, while tree branches were included as cluster factors. The Kaplan–Meier method was used to produce estimates and plots for olive drops. The Cox model was validated by checking the proportional hazard assumptions with a Schoenfeld residual analysis (Klein and Moeschberger 2006). Pairwise comparisons between treatments were performed by adjusted *P*-values (Tukey method) using the ‘emmeans’ package (Lenth et al. 2020). In damage assessment of *H. halys*, correlation tests between dislodged olives and stink bugs that were caged in each sleeve were also performed by using the ‘Spearman’ method.

Effects of insecticide applications (sprayed vs. unsprayed), olive drop over time, and their interaction were explored on overall Heteroptera and *H. halys* infestations. Linear mixed models (lme) using the ‘nlme’ package (Pinheiro et al. 2023) were employed. Insecticide application and a number of olives dropped between each sampling date were included in the model as categorical and continuous variables, respectively. Olive grove identity was included as a random factor. Residual distribution was assessed using the ‘car’ package (Fox and Weisberg 2019).

## Results

### Field Surveys of the Phytosanitary Status of Olives and of Heteroptera Community in Olive Canopies During the Early Drop Period

In 2021, all 3 investigated olive groves showed evidence of an early drop of olives. External browning and necrosis (Fig. 1A and B) were found on 41.9 ± 28.5% (mean ± SD) of dislodged olives, while the remaining ones had just some minor wilting or a slightly yellowish-green color. Once cut in half, Heteroptera feeding wounds associated with seed necrosis and lack of endosperm were found in 80.9 ± 5.5% of olives, while on 7.4 ± 4.8% of them, no symptoms were detected either inside or externally. External browning, necrosis, and Heteroptera feeding were found in all the 3 olive groves.

In 2022, 9 out of 11 investigated olive groves showed evidence of early drop of fruits (Table 2). In groves where the abnormal olive drop was detected, dislodged fruits exhibited symptoms on their surface, ranging from a slightly paler color compared to healthy ones to severe water loss and extensive pericarp that turned brown. Necrotic seeds without endosperm, endocarp whitening, and Heteroptera feeding wounds were found in 77.3 ± 17.5% of the olives. Healthy olives (those that were firmly attached to the branches) generally appeared green and turgid, with a translucent and gelatinous endosperm (Fig. 1C), and only 5.8 ± 9.3% of them presented symptoms of Heteroptera feeding activities (Table 2).

**Table 2.** Heteroptera feeding activities and stink bug community surveys performed in 2022 in 11 olive groves during early fruit development (pre-hardening of the stone) period

Code	Stink bugs					Seed necrosis with feeding mark	
	H.h.	N.v.	P.l.	P.p.	R.n.	Healthy olives (%)	Dislodged olives (%)
G1	2	0	7	0	3	0	44
G5	24	0	0	0	4	20	92
G6	7	0	0	0	0	0	86
G7	38	4	0	0	0	24	96
G8	13	1	1	4	6	1	55
G9	1	0	0	0	0	0	No olive fall
G10	10	3	0	1	0	1	84
G11	8	0	1	1	2	2	78
G12	9	0	0	4	4	1	89
G13	0	1	0	2	0	0	No olive fall
G14	11	0	0	0	5	3	72

H.h.: *Halyomorpha halys*; N.v.: *Nezara viridula*; P.l.: *Piezodorus lituratus*; P.p.: *Palomena prasina*; R.n.: *Rhaphigaster nebulosa*.

Overall, fungal colonies emerged from 2.7 ± 3.4% of seeds, 7.7 ± 7.2% of pericarps, and 42.7 ± 20.5% of petioles (Table 3), with no statistical difference between healthy and fallen olives (χ^2^ = 0.652, df = 1, *P* = 0.420). Isolation rates from petioles are statistically higher than those from seeds and pericarps (χ^2^ = 98.918, df = 2, *P* < 0.001). Pairwise comparisons are reported in Table 4. Interaction between fruit tissue and health status was not significant (*χ*^2^ = 0.676, df = 2, *P* = 0.713). The most common fungi were *Alternaria* spp. (45 colonies, 26 from dislodged olives and 19 from healthy ones), *Aureobasidium* spp. (37 colonies, 21 from dislodged olives and 16 from healthy ones), and *Phoma fungicola* (15 colonies, 5 from dislodged olives, and 10 from healthy ones). Other fungal species were less common (Table 3).

**Table 3.** Fungi emerged from seeds, pericarps, and petioles of dislodged and healthy olives collected from 11 olive groves during early fruit development (pre-hardening of the stone) period.

Fungi	Seed		Pericarp		Petiole		Total
	Dislodged	Healthy	Dislodged	Healthy	Dislodged	Healthy	
*Alternaria* spp.	1	0	4	2	21	17	45
*Aureobasidium* spp.	3	2	3	3	15	11	37
*Phoma fungicola*	0	0	1	2	4	8	15
*Nigrospora oryzae*	0	0	0	0	2	3	5
*Chaetomium* spp.	0	0	0	0	2	2	4
*Epicoccum nigrum*	0	0	2	1	0	1	4
*Sordaria* spp.	0	0	0	0	1	2	3
Others	0	0	0	0	10	5	15
Total	4	2	10	8	55	49	128
Isolation rate	4/110	2/110	10/110	7/110	48/110	46/110	117/660

**Table 4.** Pairwise comparison between health status (healthy or dislodged) and olive tissue (seed, pericarp, or petiole) on the isolation rate of fungi. The interaction term was removed since not significant (*P* > 0.05).

		Estimate (logit)	SE	Lower CI (95%)	Upper CI (95%)	Group
Health status	Healthy	−2.40	0.314	−3.02	−1.79	a
	Dislodged	−2.01	0.256	−2.51	−1.51	a
Fruit tissue	Seed	−3.732	0.463	−4.64	−2.8249	a
	Pericarp	−2.583	0.296	−3.16	−2.0026	a
	Petiole	−0.306	0.196	−0.69	0.0774	b

From sweepings, 5 species of Heteroptera were found: *H. halys*, *Nezara viridula* (Linnaeus), *Palomena prasina* (Linnaeus), *Piezodorus lituratus* (Fabricious), and *Rhaphigaster nebulosa* (Poda) (Pentatomidae). The most abundant species was *H. halys*, which was found in all the 9 olive groves where early olive drop was detected (Table 2).

### Damage Assessment of *H. halys* on Olives

**Fig. 2.**
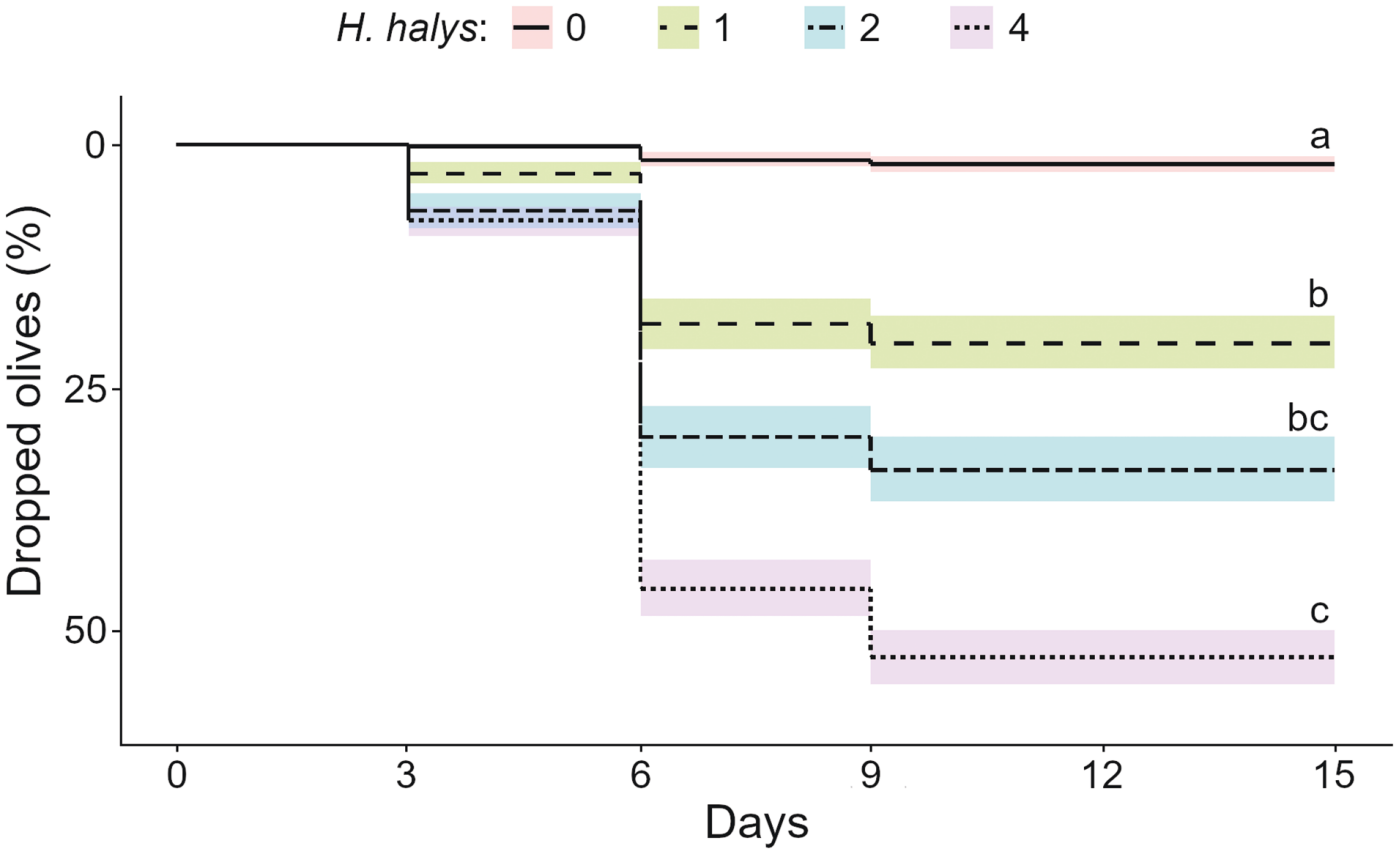
Olive drop occurring in the sleeves containing 0, 1, 2, or 4 *Halyomorpha halys* adults (7 replicates per treatment) in early fruit development (pre-hardening of the stone). Different letters indicate statistical difference between treatments (*P* = 0.05).

In the early fruit development experiment, before stink bug caging, the average number (± SD) of olives per sleeve was 155.8 ± 46.9. Olive drop in the *H. halys* infested sleeves was significantly higher compared to control (*χ*^2^ = 135.890, df = 3, *P* < 0.001; Fig. 2, Table 5) and correlated to infestation density (*r* = 0.91, *P* < 0.001). Pairwise comparisons are reported in Table 5. Wounds ascribed to stink bug feeding activities were present in 93% of the olives that fell (Table 5). The highest percentage of olive drop was 77.8%, attained with 4 *H. halys* adults.

**Table 5.** Number of dropped olives in the sleeves containing 0, 1, 2, or 4 adults of *Halyomorpha halys* and their pairwise comparisons in the early fruit development (pre-hardening of the stone) period.

N° *H. halys*	Olives	Dislodged olives				
		Total		With feeding mark		W/o feeding mark
0	157.43 ± 47.19	3.14 ± 2.73		0.71 ± 1.11		2.43 ± 1.99
1	144.00 ± 53.77	29.33 ± 16.45		26.33 ± 14.90		3.00 ± 2.19
2	154.20 ± 44.02	51.60 ± 31.63		48.20 ± 29.35		3.40 ± 3.05
4	165.29 ± 51.09	87.29 ± 32.63		84.00 ± 30.76		3.29 ± 2.29

N° *H. halys*	Estimate (log)		SE	Lower CI (95%)	Upper CI (95%)	Group
0	0		0	0	0	a
1	2.43		0.452	1.55	3.32	b
2	3.02		0.34	2.36	3.69	bc
4	3.6		0.346	2.92	4.28	c

In the late fruit development experiment, *H. halys* was not able to affect olive drop. Starting from 159.4 ± 51.3 olives per sleeve, dropped fruits in sleeves with 0, 1, 2, or 4 *H. halys* were respectively 1 ± 1.15, 0.71 ± 1.11, 0.43 ± 0.79, and 0.57 ± 0.79 (*χ*^2^ = 1.774, df = 3, *P* = 0.621). No necrosis or rots were found on the surface of infested olives. However, feeding wounds were often visible, confirmed by the presence of numerous salivary sheaths (Fig. 1H and I).

### Effectiveness of Insecticidal Strategy in Reducing Early Olive Drop and Stink Bug Population in Open Field Conditions

**Fig. 3.**
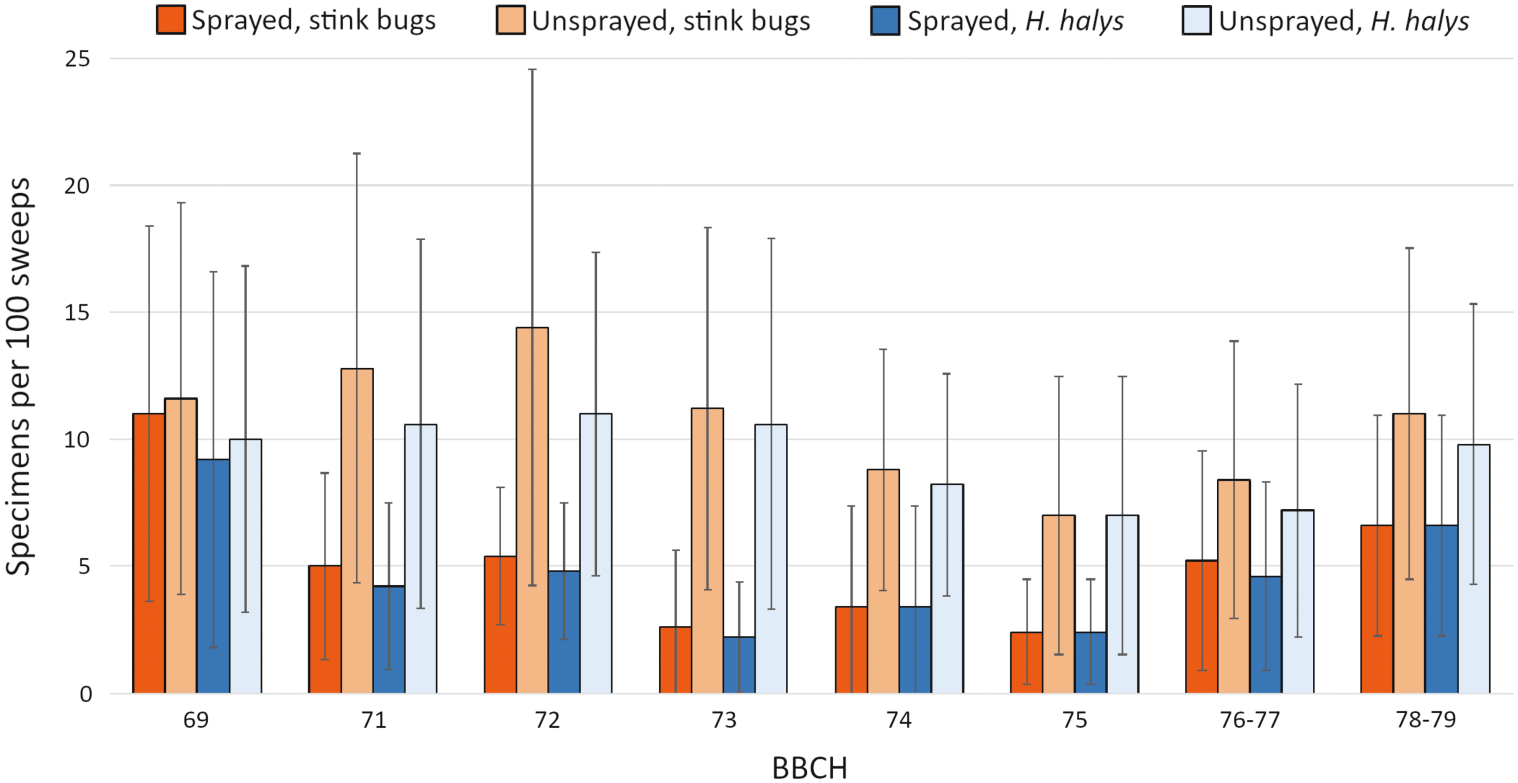
Number of stink bugs and *Halyomorpha halys* individuals found over time in plots sprayed and unsprayed with insecticides (mean of 5 olive groves). Insecticide applications were performed right after the first (BBCH69) and the third (BBCH72) sampling.

The early drop of olives was evaluated on a total of 10,048 drupes (132.21 ± 33.14 olives per branch, 2,009.60 ± 418.16 per grove). Over 98% of dropped olives fell within BBCH75 (fruits about 50% of final size, stone becomes lignified). Overall, 634 Heteroptera belonging to 6 species of stink bugs (Pentatomidae) were found, namely *H. halys* (559 specimens), *N. viridula* (49), *R. nebulosa* (20), *P. prasina* (4), *Acrosternum heegeri* Fieber (1), and *P. lituratus* (1). The assessment of Heteroptera infestation before the first insecticide application showed similar population density between the plots in each grove (total infestation *χ*^2^ = 0.081, df = 1, *P* = 0.776; *H. halys* infestation *χ*^2^ = 0.170, df = 1, *P* = 0.68) (Fig. 3).

**Fig. 4.**
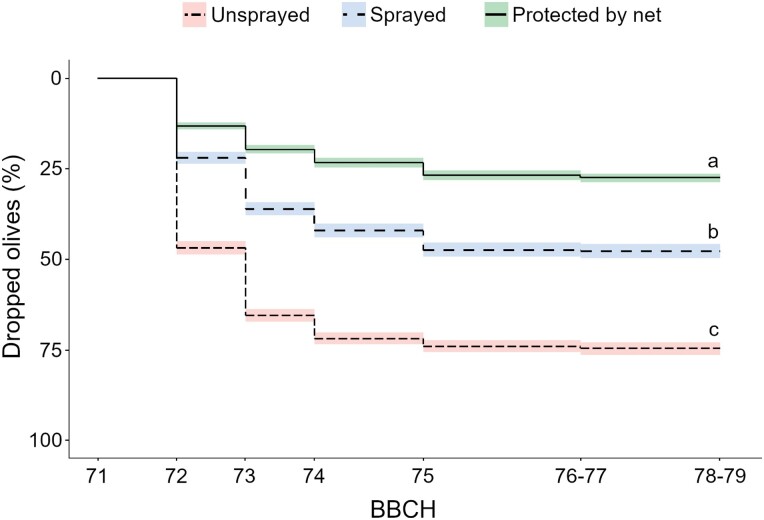
Olive drop over time in the insecticide application trials performed in 5 olive groves. The effect of olive drop of physical exclusion was included as a positive control. Different letters indicate statistical differences between treatments (*P* = 0.05).

In early fruit development, insecticide applications significantly reduced percentage of olives dropped from 74.84 ± 29.68% to 45.03 ± 30.13%, and stink bugs infestation from 9.48 ± 5.94 to 3.4 ± 2.86 stink bugs compared to unsprayed plots (Figs. 3 and 4, Table 6). Physical exclusion limited olive drop to 25.94 ± 13.67% (Fig. 4).

**Table 6.** Results from linear mixed-effect models testing insecticide applications and dropped olives against the number of *Halyomorpha halys* and total stink bug individuals found during the early fruit development (pre-hardening of the stone) period. The interaction term was removed since not significant (*P* > 0.05)

	Factors	Estimate	SE	df	*t*-value	*P*-value	
*H. halys*	Sprayed (vs. unsprayed)	−5.384426	2.1825903	8	−2.466989	0.0389	*
	Dropped olives	0.014433	0.0048859	29	2.954031	0.0062	**
Stink bugs	Sprayed (vs. unsprayed)	−5.842248	2.3562068	8	−2.479514	0.0381	*
	Dropped olives	0.024801	0.0057072	29	4.345482	0.0002	***

**Fig. 5.**
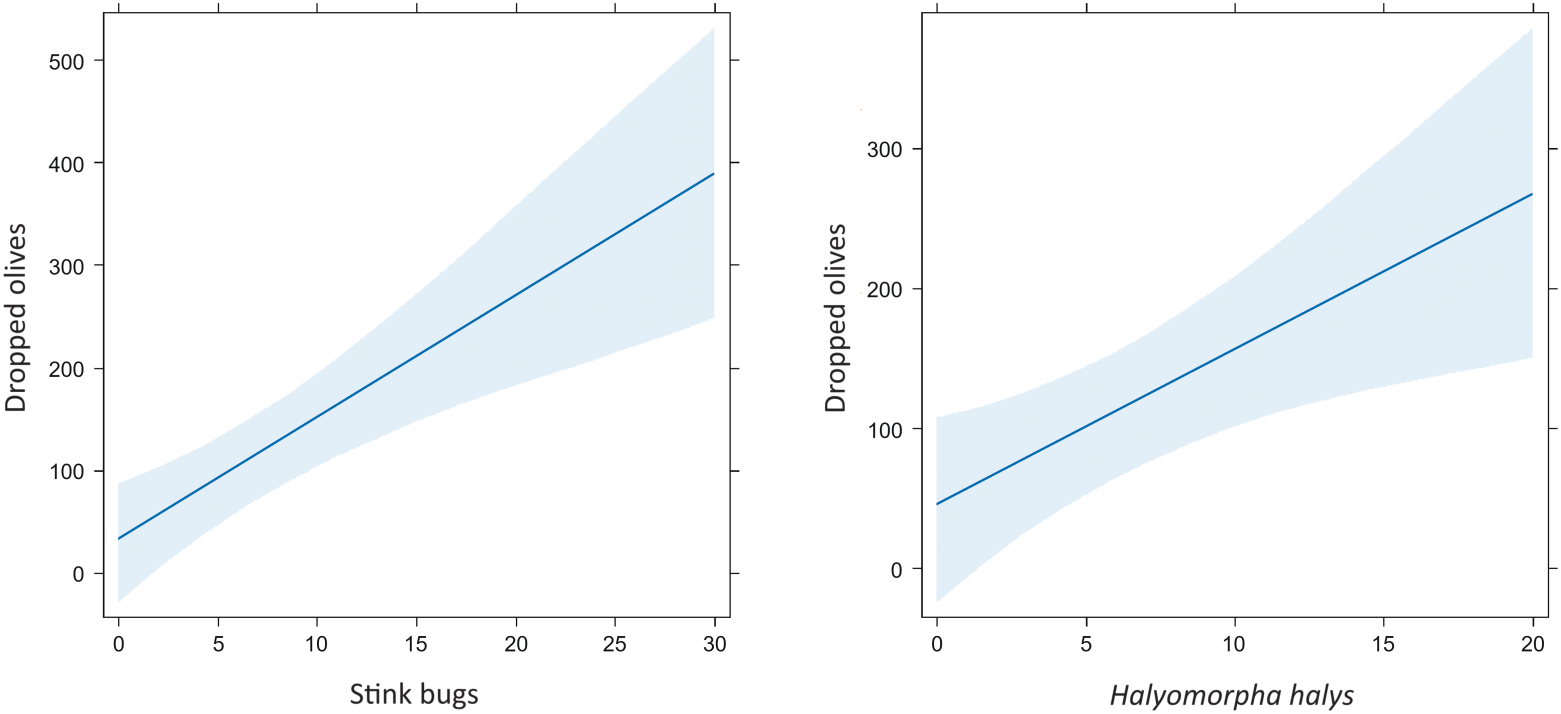
Olives dropped in response to the number of total stink bugs and *Halyomorpha halys* individuals (data from 5 samplings performed periodically from BBCH71 to BBCH75 in 5 olive groves). Model estimate (blue line) and 95% confidence interval (light blue shading) are included.

From BBCH71 to 75, a positive influence of stink bugs on early olive drop occurring outside net sleeves were found, both considering all the species together (marginal pseudo-*R*^2^ = 0.45, conditional pseudo-*R*^2^ = 0.71) and *H. halys* alone (marginal pseudo-*R*^2^ = 0.39, conditional pseudo-*R*^2^ = 0.70) (Fig. 5, Table 6).

## Discussion

The role of fungi and insects in the early olive drop disease, recently observed in olive groves located in northern Italy, was investigated. From field surveys, few fungal colonies were isolated from necrotizing seeds and dislodged olives, whilst more colonies evaded from the petioles probably because of the presence of latent fungi in the abscission wounds of flowers. The most frequently isolated fungi (i.e., *Alternaria* spp., *Aureobasidium* spp., and *Phoma fungicola*) are common endophytes (Nicoletti et al. 2020, Poveda and Baptista 2021). Although some *Alternaria* species can act as pathogens (Moral et al. 2008, Alam and Munis 2019), in our surveys *Alternaria* spp. were isolated from both healthy and dislodged olives, suggesting that they are not involved in olive drop. This can also be confirmed by the low isolation rates from seeds and pericarps, which have to be combined with the endophytic behavior of the identified fungi.

Stink bug-feeding wounds, seed necrosis, and lack of endosperm were observed in about 80% of dislodged olives and 6% of healthy ones, thus showing the existence of an association between olive drop and seed damages by insect feeding. The observed symptoms are typical of Heteroptera insects, piercing-sucking insects capable of feeding through the flesh of the fruit and reaching the seed with the mouthparts (Schaefer and Panizzi 2000). A total of 6 phytophagous Pentatomidae species were collected from olive canopies, namely *A. heegeri*, *H. halys*, *N. viridula*, *P. prasina, P. lituratus,* and *R. nebulosa.* The predominant species was *H. halys.* This invasive pest is known to feed on fruits (Acebes-Doria et al. 2016, Costi et al. 2017, Moore et al. 2019) and seeds (Hedstrom et al. 2014, Lara et al. 2017, Rijal and Gyawaly 2018), causing abortion, early fruit drop and decreased yield.

Damage assessment of *H. halys* in early and late fruit development showed that this pest can cause olive drop only in early fruit development. The number of dislodged olives per stink bug tended to decrease by increasing *H. halys* infestation and it was about 60 per week with a single insect, 55 with 2 insects and 47 with 4 insects. Maximum overall damage was achieved by a single male, which fed from 47 olives in 3 days (110 per week). The result is coherent with what was found by Daher et al. (2023) in the local olive cultivar Moraiolo, even if the number of dropped olives we recorded was higher. This may indicate a partial resistance of cv. Moraiolo to early olive drop, as well as a less susceptibility to fruit drop near the stone hardening stage. In fact, Daher et al. studies were carried out 2 wks later than our trials, close to stone hardening, probably underestimating the damage caused by *H. halys*. In early fruit development, symptoms on olives were consistent with those we found in the field and those observed by Zapponi et al. (2022), with external browning, necrosis, wilting or yellowish green color, internal seed necrosis and lack of endosperm. In addition to Zapponi et al. (2022) and Daher et al. (2023), the present study has demonstrated that early olive drop is proportional to the infestation level of *H. halys*. Notably, olive drop resulted very intense even at the lowest of tested *H. halys* infestation levels, possibly showing a relationship with the relatively low stink bugs population that can be observed in olive groves. Despite the olive drop being correlated to infestation density, olive drop per stink bug slightly decreased by increasing *H. halys* infestation, from 60 olives per week that fell with one adult to 49 olives per adult with 4 caged insects. This result might be caused by an insufficient number of fruits initially present on branches. With a damage of 60 olives per week, each *H. halys* adult would be able to drop over 300 olives from fruit set to the beginning of stone hardening (~5–6-wk period), corresponding to over 600 g at harvest considering the average weight of olives of local cultivars (Bassi 2003). The maximum fruit drop caused by a single stink bug was 47 olives in 3 days, corresponding to 110 olives per week and to over 1 kg of potential yield loss at harvest. Even if *H. halys* infestation does not affect the olive drop after stone hardening, its feeding activities reduce organoleptic quality of olive oil (Ivancic et al. 2022, Daher et al. 2023).

Field studies carried out in 2023 confirmed the results obtained in previous years. Among Heteroptera, only stink bugs were observed in olive canopies, with *H. halys* as the prevalent species (almost 90% of total stink bugs specimens). Despite stink bugs in both early and late fruit development, over 98% of abnormal olive drop occurred before the hardening of the stone, supporting the results obtained from damage assessment trials and indicating that the phenological stage plays a key role in the susceptibility to the disease. Overall, the number of olives that fell before the hardening of the stone was linked to stink bug population density, thus confirming their role in the abnormal early olive drop. With its high damage potential, greater population compared to native stink bugs, and its significant influence on olive drop when considered alone, *H. halys* is likely to be the main cause of increased fruit drop. Despite this, a contribution of other stink bugs on the early olive drop is extremely plausible, as *N. viridula* and *A. heegeri* are already reported as harmful to olive (Özgen et al. 2005, Kaul et al. 2007) and *P. lituratus* and *P. prasina* are able to cause seed damages and premature fruit drop (Saruhan and Tuncer 2010, Mutlu et al. 2018). Due to the low population of these insects in olive canopies, the early drop of olives caused by them might have always been confused with natural fruit drop. Since the establishment and population growth of *H. halys*, the scenario may have changed, with infestation densities high enough to impact the olive production, leading to substantial yield losses in olive groves. One might speculate on the connection between the occurrence of *H. halys* infestations in northern Italy—since at least 2012 (Maistrello et al. 2014)—and the subsequent emergence and escalation of the early olive drop disease. This phenomenon could be linked to the increased size of the pest population in olive-growing areas (Bariselli et al. 2016), but the correlation should be confirmed by ad hoc studies.

Notably, in southern Italy the early olive drop does not seem to be as relevant, despite the reported presence of *H. halys* (Cianferoni et al. 2018). This could be related to the low or negligible population density of brown marmorated stink bugs in these areas, due for instance to abiotic factors like temperature and relative humidity and to the landscape composition and available host plants (Laterza et al. 2022, 2023, Tamburini et al. 2023). *Halyomorpha halys* survival is indeed reduced by prolonged periods of temperatures above 30 °C, and by relative humidity below 40% (Haye et al. 2014, Scaccini et al. 2019, Khadka et al. 2020, Stahl et al. 2021). Therefore, the climate characterized by dry summers, typical of southern but not northern Italy (Beck et al. 2023), could provide unsuitable habitats for the brown stink bug, keeping its population below the damage threshold.

Insecticide applications only partially reduced stink bugs population density, leading to a proportional reduction in early olive drop. Physical exclusion of stink bugs outperformed insecticide applications, suggesting that the currently available control measures based on pesticide applications need to be improved. It should be underlined that the olive drop occurring inside the sleeves was probably due to a natural drop thinning phenomenon, which normally proceeds until about 35–45 days after full bloom (BBCH72-74) (Rallo and Fernández-Escobar 1985), and to the influence of abiotic factors (Sanzani et al. 2012).

In conclusion, this study highlights the role of stink bugs, and of *H. halys* in particular, in the early drop of olives disease. Considering the high damage potential of the brown marmorated stink bug, future research on its management is needed to efficiently reduce the early drop of olives and limit yield loss in susceptible areas, such as northern Italy. In particular, it would be desirable to develop new sustainable strategies to counteract *H. halys*.

## Data Availability

The datasets generated and/or analyzed during the current study are available from the corresponding author upon reasonable request.
